# Postmortem Microbiology in Forensic Diagnostics: Interpretation of Infectious Causes of Death and Emerging Applications

**DOI:** 10.3390/diagnostics16020325

**Published:** 2026-01-19

**Authors:** Jessika Camatti, Maria Paola Bonasoni, Anna Laura Santunione, Rossana Cecchi, Erjon Radheshi, Edoardo Carretto

**Affiliations:** 1 Department of Medicine and Surgery, University of Parma, Via Università 12, 43121 Parma, Italy; jessika.camatti@unipr.it; 2 Department of Medical and Surgical Sciences, Unit of Legal Medicine, University of Bologna, Via Irnerio 49, 40126 Bologna, Italy; 3 Pathology Unit, Azienda USL-IRCCS di Reggio Emilia, Via Amendola 2, 42122 Reggio Emilia, Italy; 4 Department of Biomedical, Metabolic and Neural Sciences, Unit of Legal Medicine, University of Modena and Reggio Emilia, Via Campi 287, 41125 Modena, Italy; annalaura.santunione@unimore.it (A.L.S.); rossana.cecchi@unimore.it (R.C.); 5 Unit of Legal Medicine and Bioethics, Azienda USL IRCCS di Reggio Emilia, Via Amendola 2, 42122 Reggio Emilia, Italy; erjon.radheshi@ausl.re.it; 6 Clinical Microbiology Laboratory, Azienda USL-IRCCS di Reggio Emilia, 42122 Reggio Emilia, Italy; edoardo.carretto@ausl.re.it; 7 U.O.C. Microbiologia e Virologia Azienda Socio Sanitaria Territoriale “Papa Giovanni XXIII”, 24127 Bergamo, Italy

**Keywords:** postmortem microbiology, forensic pathology, cause of death, sepsis, molecular diagnostics, thanatomicrobiome, postmortem interval, artificial intelligence

## Abstract

**Background/Objectives**: Postmortem microbiology has traditionally been regarded with caution in forensic practice due to concerns related to contamination, bacterial translocation, and postmortem microbial overgrowth. As a result, microbiological findings obtained after death have often been considered unreliable or of limited diagnostic value. However, growing evidence indicates that, when appropriately interpreted and integrated with autopsy findings, histopathology, and circumstantial information, postmortem microbiology can provide crucial support for cause-of-death determination. This narrative review critically examines the current role of postmortem microbiology in forensic diagnostics, with a focus on its diagnostic applications, interpretative challenges, and future perspectives. **Methods/Results:** The transition from conventional culture-based techniques to molecular approaches—including polymerase chain reaction, microbiome analysis, and metagenomic methods—is discussed, highlighting both their potential advantages and inherent limitations within the forensic setting. Particular attention is devoted to key interpretative issues such as postmortem interval, sampling strategies, contamination, and bacterial translocation. In addition to cause-of-death attribution, emerging applications—including postmortem interval estimation, trace evidence analysis, and artificial intelligence–based models—are reviewed. Although these approaches show promising research potential, their routine forensic applicability remains limited by methodological heterogeneity, lack of standardization, and interpretative complexity. **Conclusions:** In conclusion, postmortem microbiology represents a valuable diagnostic tool when applied within a multidisciplinary forensic framework. Its effective use requires cautious interpretation and integration with pathological and contextual evidence, avoiding standalone or automated conclusions. Future progress will depend on standardized methodologies, multidisciplinary collaboration, and a clear distinction between experimental research and routine forensic practice.

## 1. Introduction

Postmortem microbiology (PMM) has long represented a controversial and, at times, underestimated component of forensic investigations. Traditionally regarded with skepticism due to concerns about postmortem contamination, bacterial translocation, and microbial overgrowth, microbiological findings obtained after death have often been considered unreliable or of limited diagnostic value. As a consequence, PMM has historically been underutilized in routine forensic practice, particularly when compared with its central role in clinical diagnostics [1,2,3].

However, this cautious attitude has also led, in some cases, to the loss of potentially crucial diagnostic information, especially in deaths related to infections, sepsis, or rapidly progressive disease processes [4,5]. In the forensic setting, the challenge is not merely to detect microorganisms after death, but to correctly interpret their presence within a complex biological and temporal context, requiring cautious forensic interpretation [6,7].

### 1.1. From Classical Postmortem Cultures to the Microbiome Era

Early applications of PMM were largely based on conventional culture techniques, primarily aimed at identifying pathogens responsible for fatal infections. While culture-based approaches remain valuable, they are inherently limited by postmortem changes, fastidious organisms, prior antimicrobial therapy, and difficulties in distinguishing contamination from true infection [1,2,3].

Over the last two decades, advances in molecular biology have profoundly reshaped the field. Polymerase chain reaction (PCR), broad-range 16S rRNA gene sequencing, and, more recently, metagenomic approaches have enabled culture-independent detection of microorganisms, expanding the spectrum of detectable pathogens and allowing a more comprehensive characterization of postmortem microbial communities [8,9,10].

Beyond bacterial profiling, molecular forensic microbiology has progressively expanded toward non-bacterial targets. Sequencing approaches targeting the 18S rRNA gene and internal transcribed spacer (ITS) regions have been increasingly applied for the postmortem detection of fungal pathogens, particularly in immunocompromised individuals and in cases of invasive mycoses. Similarly, virome-oriented approaches, including targeted viral PCR assays and metagenomic sequencing, have enabled the identification of viral agents in postmortem tissues, broadening the diagnostic spectrum of PMM beyond a purely bacteriocentric framework [10].

These advances have reinforced the role of molecular methods in detecting fastidious, uncultivable, or partially treated pathogens; however, they also introduce interpretative challenges analogous to those observed for bacterial targets, including contamination, postmortem redistribution, and the inability to distinguish between latent presence and true antemortem infection [10].

Within this context, the concepts of “thanatomicrobiome”and “necrobiome” have emerged, describing the dynamic microbial communities associated with internal organs, body surfaces, and the surrounding environment after death [9,11]. These studies have demonstrated that postmortem microbial communities are not random, but are shaped by a combination of deterministic factors—such as anatomical site, postmortem interval, and environmental conditions—and stochastic processes, resulting in partially predictable and probabilistic patterns rather than fixed microbial trajectories.

### 1.2. Diagnostic Value and Interpretative Complexity in Forensic Settings

Despite growing scientific interest, the translation of PMM into routine forensic diagnostics remains challenging. Unlike clinical microbiology, forensic investigations must address questions related to causality, timing, and legal responsibility, often in the absence of antemortem clinical data. In this setting, the mere detection of microorganisms does not equate to proof of infection or cause of death [3,7,12].

Recent forensic case reports and series have highlighted the diagnostic contribution of PMM in identifying fatal infections, including bacterial sepsis, meningitis, myocarditis, and viral diseases that were clinically unrecognized before death [13,14,15,16]. These cases emphasize that, when appropriately interpreted, postmortem microbiological findings can play a decisive role in cause-of-death attribution, particularly in sudden or unexpected deaths.

At the same time, the increasing use of high-throughput sequencing and AI-based analytical tools has generated both enthusiasm and concern. While machine learning models have shown promising results in PMI estimation and pattern recognition, their forensic applicability is still limited by lack of standardization, small datasets, and interpretative opacity [17,18,19,20].

### 1.3. Aim and Scope of the Review

The aim of this narrative review is to critically examine the current role of PMM in forensic diagnostics, focusing on its diagnostic value, interpretative challenges, and realistic future perspectives. Rather than providing a systematic synthesis of the literature, this review integrates experimental studies, consensus documents, reviews, and illustrative forensic case reports to highlight strengths, limitations, and unresolved issues in the field.

### 1.4. Search Strategy and Methodological Approach

This narrative review was based on a focused literature search conducted primarily in PubMed, aimed at identifying studies addressing PMM and its role in forensic diagnostics. The search covered publications from approximately the last 15 years, with particular emphasis on studies published in the last decade. Search terms included combinations of “*PMM*”, “*forensic microbiology*”, “*thanatomicrobiome*”, “*necrobiome*”, “*sepsis*”, “*infection*”, and “*cause of death*”.

Original research articles, narrative and systematic reviews, consensus statements, and illustrative forensic case reports were considered. Only studies written in English, with full text available, and involving human cases were included. Animal studies, purely experimental models without direct forensic relevance, and articles lacking diagnostic or interpretative implications were excluded.

Study selection was guided by relevance to forensic diagnostic practice, with particular emphasis on cause-of-death attribution, sampling strategies, methodological limitations, and interpretative challenges. Given the substantial heterogeneity in study design, biological matrices, analytical methods, and forensic scenarios, a systematic review approach was considered inappropriate. Accordingly, a critical narrative synthesis was adopted to integrate current evidence with a forensic interpretative perspective. No predefined number of articles was therefore targeted, and literature selection was guided by thematic relevance and diagnostic significance rather than by predefined quantitative thresholds. Particular attention is given to the interpretation of microbiological findings in relation to cause-of-death determination, the impact of PMI and sampling strategies, and the emerging role of molecular and computational approaches. By adopting a diagnostic and forensic-oriented perspective, this review aims to support pathologists and forensic practitioners in the informed and critical use of PMM in routine practice.

## 2. PMM in Forensic Practice

### 2.1. Indications for Postmortem Microbiological Investigations

In forensic pathology, postmortem microbiological investigations should be regarded as a targeted diagnostic tool rather than a routine or indiscriminate procedure [21]. Their primary indication lies in cases in which an infectious cause of death is suspected or cannot be reasonably excluded on the basis of macroscopic findings alone [4]. These include suspected sepsis, rapidly progressive infections, meningitis or encephalitis, myocarditis, and sudden unexpected deaths with nonspecific autopsy findings [5,6].

PMM is particularly valuable in deaths occurring outside hospital settings, in individuals with limited or absent antemortem clinical data, and in vulnerable populations such as immunocompromised patients, splenectomized individuals, and children [4,21]. In these contexts, microbiological analyses may provide decisive information for cause-of-death attribution and medico-legal evaluation [22,23]. The main forensic applications of PMM, together with their diagnostic aims, commonly analyzed samples, and principal limitations, are summarized in Table 1.

Conversely, the indiscriminate use of PMM without a clear diagnostic question increases the risk of misleading results and overinterpretation [3]. For this reason, several authors and consensus documents have emphasized the importance of a hypothesis-driven approach, in which microbiological investigations are planned and interpreted within the overall forensic framework [12,24].

### 2.2. Biological Samples and Target Sites

The diagnostic value of PMM is strongly influenced by the selection of appropriate biological samples. Traditionally, blood and cerebrospinal fluid have been considered the specimens of choice; however, their interpretation is complicated by PMBT and contamination, particularly with increasing PMI [2,3,24].

According to ESGFOR recommendations, blood sampling for postmortem microbiological investigations should preferentially be performed from peripheral sites such as the femoral or subclavian vessels, using strict aseptic techniques to reduce the risk of contamination and postmortem bacterial translocation [24]. Similarly, ESGFOR guidelines recommend that cerebrospinal fluid be collected under controlled conditions, preferably via cisternal or lumbar puncture, in order to minimize contamination and improve the interpretative reliability of postmortem findings [24].

Sampling of deep organs such as spleen, liver, lung, heart, and brain has been proposed as a complementary strategy, as these sites may better reflect systemic infection when collected under controlled conditions [3,8,24]. In addition, alternative biological fluids, including pericardial and pleural effusions, have shown potential diagnostic value, especially in suspected sepsis or inflammatory conditions [6].

Regardless of the sampling site, strict aseptic techniques and standardized protocols are essential to minimize contamination. International consensus statements highlight that improper sampling may compromise even the most advanced analytical methods, ultimately undermining forensic interpretation [24,25].

### 2.3. Culture-Based and Molecular Approaches

Culture-based methods remain a cornerstone of PMM due to their availability, low cost, and ability to provide viable isolates for further characterization [1,2]. When positive results are obtained from multiple concordant samples and supported by histopathological evidence, cultures may significantly contribute to cause-of-death determination [7].

However, culture techniques are limited by postmortem changes, prior antimicrobial therapy, and the overgrowth of commensal or environmental microorganisms, which may complicate interpretation and reduce diagnostic sensitivity [1,3]. These limitations have driven the increasing use of molecular approaches, including targeted PCR assays and broad-range sequencing techniques, which allow the detection of fastidious, uncultivable, or viral pathogens in the postmortem setting [10,12,26,27].

For culture-based methods, the diagnostic yield is strongly influenced by postmortem interval and by antemortem antimicrobial therapy. Early postmortem sampling, when feasible, is generally associated with higher reliability in infection-related deaths, whereas prolonged PMI favors microbial overgrowth, translocation, and loss of pathogen viability. In addition, prior antibiotic exposure may significantly reduce culture sensitivity, further increasing interpretative uncertainty. Consequently, the concept of an ‘effective time window’ for postmortem sampling should be regarded as case-dependent rather than fixed, and culture results must be interpreted cautiously, particularly in cases with prolonged PMI or prior antimicrobial exposure [3,7].

Molecular approaches in PMM are not limited to bacterial detection. Targeted PCR assays and sequencing strategies addressing fungal (18S rRNA, ITS regions) and viral genomes have gained increasing relevance in forensic casework, particularly in the investigation of fatal myocarditis, meningoencephalitis, and opportunistic infections. These methods may prove decisive in cases where conventional cultures are negative or unavailable, or when antemortem antimicrobial therapy has been administered [26,27].

Nevertheless, the forensic interpretation of non-bacterial molecular findings requires the same level of caution applied to bacterial targets. The detection of fungal or viral nucleic acids in postmortem samples does not, per se, establish causality and requires careful interpretation in light of postmortem biological dynamics and potential confounding factors. As with bacterial sequencing, these techniques primarily indicate the presence of microbial genetic material and should therefore be interpreted within a comprehensive forensic framework rather than as standalone diagnostic evidence [26,27].

High-throughput sequencing and microbiome-based analyses further expand the diagnostic spectrum, but they also introduce new interpretative challenges. Molecular detection reflects the presence of microbial genetic material and, in the postmortem setting, requires careful interpretation in the postmortem setting, considering the risk of overinterpretation [3,10,12]. For this reason, current evidence supports an integrated approach in which culture-based and molecular methods are used complementarily, rather than as mutually exclusive alternatives.

### 2.4. Sampling Strategies and Timing

The reliability of postmortem microbiological findings is critically dependent on sampling strategies and timing. In contrast to clinical microbiology, forensic sampling is often performed hours or days after death, under conditions that may favor microbial translocation and postmortem proliferation [28,29]. Consequently, the PMI represents a major confounding factor that must be explicitly considered when planning and interpreting microbiological investigations [30,31].

Early postmortem sampling, when feasible, is generally associated with a higher diagnostic yield and lower risk of contamination. Several authors have emphasized that microbiological investigations performed within a short PMI are more likely to reflect antemortem infection rather than postmortem microbial dynamics [32,33,34]. However, in routine forensic practice, early sampling is not always possible, particularly in cases discovered late or in non-hospital settings.

The timing of sampling should therefore be documented and interpreted as an integral part of the diagnostic process. Rather than representing an absolute contraindication, a prolonged PMI should prompt a more cautious interpretation, emphasizing concordance across multiple samples and consistency with postmortem dynamics [35,36,37].

### 2.5. Quality Assurance and Standardization in Forensic Microbiology

Quality assurance represents a central issue in PMM, where variability in sampling, laboratory procedures, and interpretative criteria can significantly influence results. Unlike clinical microbiology, forensic investigations lack universally adopted standard operating procedures, resulting in heterogeneous practices across institutions and jurisdictions [38,39].

International working groups, such as the ESGFOR, have proposed consensus-based recommendations addressing critical aspects of PMM, including sampling techniques, choice of specimens, and documentation of PMI. These guidelines underline that methodological rigor is a prerequisite for meaningful interpretation, regardless of the analytical technique employed [24,25].

Standardization is particularly relevant when molecular methods and high-throughput sequencing are applied. Differences in DNA extraction protocols, target regions, sequencing platforms, and bioinformatic pipelines may lead to substantially different microbial profiles, even when identical samples are analyzed [19,40]. In the forensic context, such variability poses significant challenges for reproducibility and legal defensibility.

For these reasons, PMM should be embedded within a structured quality framework that includes standardized sampling protocols, transparent reporting of methods, and structured interpretative evaluation of results. Without such safeguards, even technically sophisticated analyses risk producing findings that are scientifically intriguing but diagnostically misleading.

**Figure 1:**
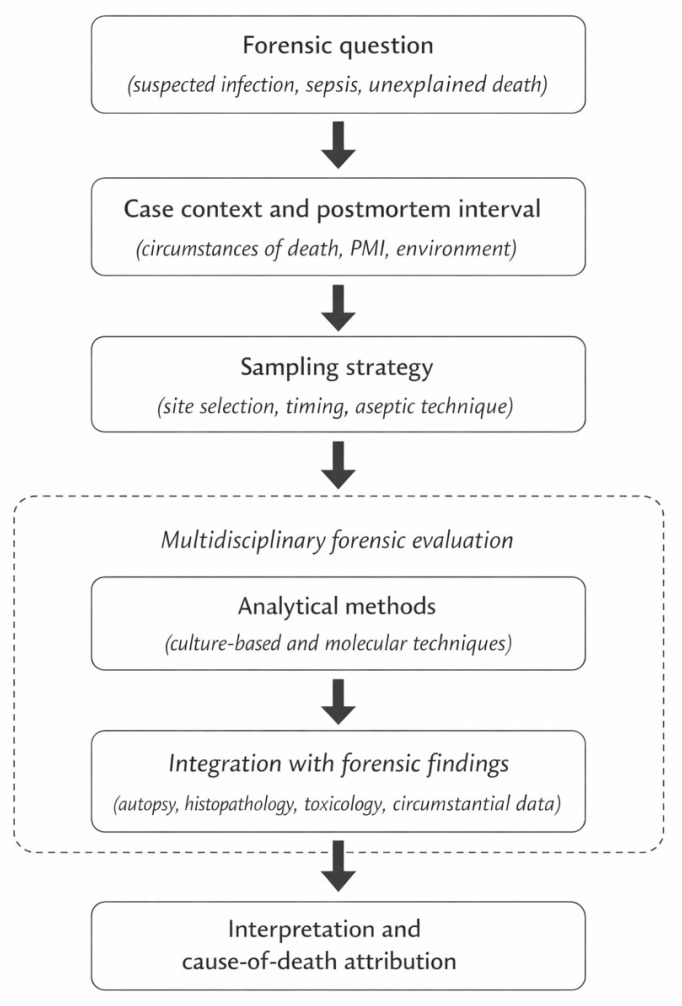
Workflow of PMM in forensic diagnostics.

As summarized in Figure 1, the diagnostic value of postmortem microbiological findings ultimately depends on their coherence with the overall forensic context and on careful interpretative evaluation, rather than on isolated microbiological results [13,14,15,16,41,42,43,44,45,46,47,48,49,50,51,52].

## 3. Cause-of-Death Attribution: Diagnostic Value of PMM

### 3.1. Sepsis and Systemic Infections

Sepsis represents one of the most challenging conditions for postmortem diagnosis and a major indication for forensic microbiological investigations. In contrast to clinical settings, where sepsis is diagnosed on the basis of dynamic clinical, laboratory, and microbiological parameters, forensic investigations must often rely on static postmortem findings, frequently in the absence of detailed antemortem information [1,2].

PMM may provide critical support in identifying systemic infections, particularly when supported by evidence of systemic inflammatory response. Several forensic studies have demonstrated that microbiological findings, when concordant across multiple sampling sites and supported by pathological findings, can significantly contribute to cause-of-death attribution in suspected sepsis [2,6,21].

However, the interpretation of positive microbiological results in suspected sepsis is inherently complex. PMBT from the gastrointestinal tract and postmortem proliferation may lead to false-positive findings, especially with increasing PMI. For this reason, the detection of microorganisms in blood or tissues requires cautious interpretation due to postmortem bacterial translocation and proliferation [2,3,24].

Illustrative forensic cases have shown that early postmortem sampling and multi-site concordance increase the likelihood that microbiological findings reflect true antemortem infection. Conversely, discordant or isolated positive results, particularly when supportive pathological features are lacking, should be interpreted with caution [22].

### 3.2. Central Nervous System Infections

Infections of the central nervous system, including meningitis and encephalitis, represent another important diagnostic domain for PMM. These conditions may cause rapid clinical deterioration and sudden death, sometimes without clear macroscopic findings at autopsy. In such cases, postmortem microbiological investigations of cerebrospinal fluid and brain tissue may be essential for identifying the underlying cause of death [33,45,53].

Molecular methods have expanded the diagnostic possibilities in postmortem CNS infections, allowing the detection of fastidious or partially treated pathogens. PCR-based assays and broad-range sequencing have proven particularly useful when conventional cultures are negative or inconclusive, although their results require careful interpretation in light of potential contamination and postmortem changes [33,45,52,53,54].

The diagnostic value of PMM in CNS infections is maximized when microbiological findings are supported by concordant pathological features, such as meningeal inflammation or encephalitic changes. In the absence of such correlation, the detection of microbial DNA alone should not be considered sufficient to establish causality [21].

### 3.3. Cardiac Infections and Myocarditis

Cardiac infections, particularly myocarditis, are a well-recognized cause of sudden unexpected death and pose significant diagnostic challenges in forensic pathology. Macroscopic findings may be subtle or absent, and histological changes can be focal or nonspecific. In this context, PMM and molecular virology may play a decisive role in identifying infectious etiologies [15,23,55].

Recent forensic case reports have demonstrated the contribution of postmortem microbiological and molecular investigations in diagnosing fatal viral myocarditis, including cases caused by Epstein–Barr virus and other cardiotropic viruses. These cases highlight the importance of extending PMM beyond bacterial pathogens and adopting a broader infectious perspective [15].

Nevertheless, as with other forensic applications, the detection of viral genomes in cardiac tissue does not automatically imply causation. Interpretation requires careful evaluation of inflammatory patterns and exclusion of alternative causes of death. This integrated approach is essential to avoid overinterpretation and to ensure diagnostic robustness in medico-legal contexts [21].

### 3.4. Viral and Non-Bacterial Infections in Forensic Contexts

Although bacterial infections have traditionally dominated postmortem microbiological investigations, viral and other non-bacterial pathogens are increasingly recognized as significant contributors to sudden and unexpected deaths. Advances in molecular diagnostics have facilitated the detection of viral agents in postmortem samples, broadening the scope of forensic microbiology [52,53].

Systematic reviews and case-based studies have shown that viral infections may be underdiagnosed causes of sudden non-cardiac death, particularly in young individuals and in cases with limited antemortem symptoms. PMM, when combined with histological and immunohistochemical analyses, can therefore provide crucial insights into otherwise unexplained fatalities [15,53].

These findings underscore the need to move beyond a purely bacteriocentric approach in forensic microbiology. A comprehensive diagnostic strategy should consider bacterial, viral, and, when appropriate, fungal pathogens, tailored to the specific forensic scenario and supported by careful forensic interpretation [14,16,21].

## 4. Interpretation Challenges in PMM

The interpretative challenges discussed in this section primarily reflect PMI-dependent biological changes affecting postmortem microbial findings. Microbial overgrowth, translocation, and redistribution represent interconnected consequences of postmortem biological processes, whose relative contribution varies according to PMI, sampling site, acnd environmental conditions [3,41]. For clarity, these phenomena are discussed separately, as they arise from distinct mechanisms and have different implications for forensic interpretation.

### 4.1. PMI and Microbial Overgrowth

The PMI represents one of the most critical variables affecting the interpretation of microbiological findings after death. Following cessation of circulation and immune function, endogenous microbial communities undergo profound changes driven by tissue autolysis, hypoxia, and loss of physiological barriers. These processes promote microbial proliferation and redistribution, complicating the distinction between antemortem infection and postmortem microbial overgrowth [56,57,58,59,60].

Numerous studies on the thanatomicrobiome have demonstrated that microbial populations increase and shift in composition as a function of time after death, with certain taxa—such as anaerobic bacteria—becoming increasingly dominant. Importantly, this postmortem expansion does not necessarily reflect pathological processes that were active before death, but rather predictable biological dynamics occurring during decomposition [56,57,58,59,60,61].

From a forensic diagnostic perspective, this phenomenon poses a substantial interpretative challenge. The presence of high bacterial loads or the detection of microorganisms typically associated with infection may lead to overinterpretation if PMI-related changes are not adequately considered. This is particularly relevant when molecular methods with high analytical sensitivity are employed, as they may detect microbial DNA even in the absence of viable organisms or active infection [56,59,60].

Accordingly, PMI should not be regarded merely as contextual information, but as a central interpretative variable that directly influences the diagnostic weight of microbiological findings. Short PMI intervals generally increase the likelihood that detected microorganisms reflect antemortem conditions, whereas prolonged intervals necessitate a more cautious interpretative approach [22].

### 4.2. Bacterial Translocation and Postmortem Redistribution

Bacterial translocation from the gastrointestinal tract to normally sterile tissues is a well-documented postmortem phenomenon and a major source of diagnostic uncertainty in forensic microbiology. Following death, the breakdown of mucosal barriers and vascular integrity facilitates the passive spread of enteric microorganisms into the bloodstream and internal organs, particularly liver, spleen, and lungs [2].

This redistribution may occur relatively early after death and is influenced by factors such as PMI, body position, environmental temperature, and pre-existing pathological conditions. As a result, the detection of enteric bacteria in postmortem blood or tissue samples does not automatically indicate systemic infection or sepsis [2,3,57,60].

Several forensic studies have emphasized that bacterial translocation can produce microbiological patterns that mimic antemortem bacteremia, especially when sampling is delayed or performed without strict aseptic precautions. This phenomenon underscores the importance of interpreting microbiological results in relation to evidence of tissue inflammation and host response, rather than relying on microbiology alone [24,25].

The challenge of distinguishing translocation from true infection is further compounded by the use of molecular techniques, which detect bacterial DNA regardless of organism viability. Consequently, molecular positivity in the absence of supportive inflammatory or tissue changes should be interpreted as evidence of microbial presence rather than proof of causation [2,33,61].

The detection of multiple microorganisms in postmortem samples represents a frequent and challenging finding in PMM. Polymicrobial results may arise from postmortem bacterial translocation, environmental contamination, or overgrowth of commensal flora, particularly with increasing postmortem interval [3,7].

In forensic diagnostics, the identification of multiple microbial species rarely supports a direct causal role in death unless corroborated by concordant findings across multiple sampling sites and supportive evidence of tissue invasion or host response. In the absence of such correlation, polymicrobial findings should be interpreted cautiously and are more suggestive of postmortem processes than of true antemortem infection [3,7].

### 4.3. Contamination During Sampling and Laboratory Processing

Contamination represents another major interpretative pitfall in PMM and may occur at multiple stages, including sample collection, transport, and laboratory processing. In forensic settings, sampling is often performed under less controlled conditions than in clinical environments, increasing the risk of introducing exogenous microorganisms [12].

Improper sampling techniques, reuse of instruments, or inadequate disinfection may result in false-positive findings that can be difficult to distinguish from genuine postmortem or antemortem microbial presence. This risk is particularly pronounced for low-biomass samples, such as blood or cerebrospinal fluid, where even minimal contamination can have significant interpretative consequences [24].

Consensus guidelines stress that methodological rigor during sampling is essential to preserve the diagnostic value of PMM. Documentation of sampling conditions, use of sterile instruments, and collection of multiple samples for concordance assessment are critical components of quality assurance [25,32].

From an interpretative standpoint, isolated positive results, especially when inconsistent with other microbiological data or expected postmortem patterns, should raise suspicion of contamination. In contrast, reproducible findings across independent samples and methods provide stronger support for diagnostic relevance [1,2].

### 4.4. Colonization Versus Infection: The Problem of Causality

One of the most fundamental challenges in PMM is distinguishing colonization from true infection. The detection of microorganisms in postmortem samples does not inherently imply pathogenicity or causal involvement in death. Many microorganisms detected after death may represent commensal flora, opportunistic colonizers, or postmortem proliferators rather than etiological agents [3,60].

This issue is particularly relevant in the era of high-throughput sequencing, where comprehensive microbial profiles can be generated from virtually any tissue. While such data provide valuable insights into postmortem microbial dynamics, they also increase the risk of overinterpretation, especially when forensic conclusions are drawn without adequate pathological correlation [62,63,64].

Forensic causality requires more than microbial detection. Evidence of tissue invasion, host inflammatory response, and anatomical plausibility must be demonstrated to support an infectious cause of death. In the absence of these elements, microbiological findings should be regarded as contributory or contextual rather than determinative [21].

Several forensic case reports illustrate that microbiological findings acquire diagnostic significance only when embedded within a coherent pathological narrative. This principle highlights the need for cautious forensic interpretation and avoidance of microbiology-driven conclusions in isolation [13,15].

### 4.5. Interpretation of Molecular and AI-Based Results

The increasing application of molecular techniques and AI–based analytical tools introduces additional interpretative challenges in forensic microbiology. While machine learning models have demonstrated promising capabilities in pattern recognition and classification tasks, their forensic validity remains limited by methodological heterogeneity, small datasets, and lack of external validation [63,64,65,66].

AI-based predictions, particularly those related to PMI estimation or cause-of-death inference, may appear objective but are inherently dependent on the quality and representativeness of the training data. In forensic contexts, where biological variability and environmental factors are substantial, overreliance on algorithmic outputs may lead to misleading conclusions [63,64].

Therefore, molecular and AI-derived results should be interpreted as supportive tools rather than definitive evidence. Their integration into forensic practice requires transparency, validation, and critical appraisal, with ultimate responsibility resting on the forensic pathologist rather than the analytical platform [54].

### 4.6. Methodological and Technological Limitations Affecting Evidentiary Weight

Beyond biological postmortem changes, the intrinsic characteristics and limitations of analytical technologies substantially affect the evidentiary weight of PMM findings [3]. Culture-based methods allow viability assessment and antimicrobial susceptibility testing but are highly sensitive to PMI, antemortem antibiotic therapy, and sample contamination. In contrast, molecular techniques, including PCR and sequencing-based approaches, offer higher analytical sensitivity and the ability to detect fastidious or non-cultivable microorganisms, but do not provide information on microbial viability and may detect residual or clinically irrelevant genetic material.

These methodological differences have direct implications for forensic interpretation. Highly sensitive molecular methods increase the risk of overinterpretation in the absence of supportive pathological correlation, while negative culture results do not exclude infection in cases of prolonged PMI or prior antimicrobial exposure [7]. Accordingly, the evidentiary value of PMM findings depends not only on the detected microorganism but also on the analytical approach employed and its inherent limitations.

A comparative overview of laboratory detection methods and computational analytical approaches, including their respective strengths and limitations, is provided in Table 2.

## 5. Beyond Cause of Death: Emerging Forensic Applications

### 5.1. Thanatomicrobiome and PMI Estimation

The analysis of postmortem microbial communities has generated considerable interest as a potential tool for PMI estimation. Studies on the thanatomicrobiome have demonstrated that microbial populations undergo temporal changes after death, following patterns that may be partially predictable under controlled conditions [58,59].

Experimental and observational studies have reported associations between PMI and shifts in microbial composition across different anatomical sites, including internal organs, oral cavity, skin, and surrounding environments. These findings have supported the concept of a “microbial clock,” suggesting that postmortem microbial succession could complement traditional PMI estimation methods [11,61].

However, despite promising experimental results, the forensic applicability of microbiome-based PMI estimation remains limited. Microbial succession is influenced by numerous confounding variables, including temperature, humidity, body position, cause of death, medical history, and environmental exposure. As a result, interindividual variability often exceeds temporal trends, particularly in real-world forensic scenarios [29,67].

Consequently, current evidence supports the use of thanatomicrobiome data as a research tool or adjunctive indicator rather than a standalone method for PMI estimation. Its role in forensic practice should be considered exploratory, requiring further validation and standardization before routine application.

### 5.2. Trace Evidence and Forensic Identification

Beyond PMI estimation, microbial analysis has been explored as a potential source of trace evidence in forensic investigations. Human-associated microbiomes, particularly those of the skin, oral cavity, and other exposed surfaces, may reflect individual- or environment-specific characteristics that could assist forensic identification [68,69,70,71].

Studies have suggested that microbial signatures can persist on personal objects, clothing, or environmental substrates, allowing probabilistic association between individuals and locations. Such approaches have been investigated in contexts including contact tracing, personal object handling, and geolocation inference [50,72].

Despite these intriguing findings, significant limitations remain. Microbial profiles are highly dynamic and susceptible to environmental influences, temporal variation, and methodological bias. Moreover, the absence of standardized databases and reference frameworks currently precludes robust forensic attribution based solely on microbiome evidence [50].

Accordingly, microbiome-based trace evidence should be regarded as supportive information rather than definitive identification evidence. Its forensic value lies primarily in supporting or excluding investigative hypotheses rather than providing conclusive individual attribution.

### 5.3. Environmental and Context-Specific Applications

Microbial analysis has also been applied to specific forensic contexts, including drowning investigations, soil and water exposure, and environmental reconstruction. Studies examining bacterial communities in aquatic environments have demonstrated that microbial profiles may assist in inferring drowning sites or postmortem immersion conditions [73].

Similarly, environmental microbiome studies have explored the potential of microbial succession in soil, vegetation, and confined spaces to provide contextual information regarding body deposition and postmortem movement [57,67]. These approaches emphasize the ecological dimension of forensic microbiology, extending analysis beyond the body itself.

While environmentally informed microbiological investigations offer valuable contextual insights, their interpretative complexity and susceptibility to confounding factors currently limit their evidentiary weight. As with other emerging applications, environmental microbiome data should be interpreted cautiously within the forensic context.

### 5.4. Mycobiome, Volatile Compounds, and Non-Bacterial Targets

Recent research has expanded forensic microbiology beyond bacterial communities to include fungal populations and microbial metabolites. Studies on the postmortem mycobiome suggest that fungal succession may provide additional information, particularly in advanced stages of decomposition where bacterial signals become less informative [74].

In parallel, microbial volatile organic compounds (VOCs) have been investigated as potential indicators of decomposition stage and postmortem processes. VOC profiling reflects microbial metabolic activity and may contribute to postmortem timing or environmental assessment [75].

Although these approaches remain largely experimental, they highlight the multifaceted nature of postmortem microbial activity and underscore the potential for integrating multiple biological signals into future forensic investigations. At present, however, their application is best confined to research settings rather than routine forensic diagnostics.

## 6. AI and Advanced Molecular Methods

### 6.1. Machine Learning and AI in Forensic Microbiology

The application of AI and machine learning techniques to forensic microbiology has expanded rapidly in recent years, driven by the increasing availability of high-dimensional microbiome datasets. Several studies have explored the use of supervised and unsupervised algorithms to identify microbial patterns associated with PMI, cause of death, or environmental context [63,64,65]. However, the forensic applicability of these models remains limited by multiple factors, including small sample sizes, lack of external validation, and sensitivity to confounding variables such as temperature, sampling site, and individual biological variability [29].

A further concern relates to interpretability. Many machine learning models function as “black boxes,” producing outputs that are difficult to translate into biologically or pathologically meaningful explanations. In forensic contexts, where transparency and reproducibility are essential for legal admissibility, this lack of interpretability represents a significant limitation [50].

Consequently, AI-based tools should currently be regarded as exploratory or supportive instruments rather than standalone diagnostic solutions. Their integration into forensic practice requires rigorous validation, standardized workflows, and clear communication of uncertainties.

### 6.2. High-Resolution Sequencing and Multi-Omics Approaches

Advances in sequencing technologies have enabled increasingly detailed characterization of postmortem microbial communities. Beyond traditional 16S rRNA gene sequencing, shotgun metagenomics and emerging approaches such as 2bRAD-M sequencing offer higher taxonomic resolution and functional insights [76]. While shotgun metagenomics provides broader coverage and functional information, it is associated with higher costs, greater computational demands, and increased risk of environmental contamination [19,40,76].

Multi-omics approaches, integrating genomic, transcriptomic, and metabolomic data, represent a promising avenue for future research. However, their complexity and limited standardization currently restrict their use to experimental settings. In routine forensic diagnostics, simpler and well-validated methods remain preferable, provided they are interpreted cautiously in the forensic setting.

The main analytical approaches currently used in PMM, together with their respective strengths, limitations, and forensic applicability, are summarized in Table 3.

## 7. Guidelines, Standardization, and Current Gaps

### 7.1. Existing Guidelines and Consensus Statements

Several international working groups have addressed the challenges of PMM by proposing consensus-based recommendations for sampling, analysis, and interpretation. Among these, the ESGFOR guidelines represent a significant effort to harmonize forensic microbiological practices across institutions and jurisdictions [24,25].

These documents emphasize the importance of hypothesis-driven investigations, standardized sampling techniques, documentation of PMI, and structured interpretative approaches. They also highlight the need to adapt microbiological methods to the forensic context rather than directly transferring clinical protocols.

### 7.2. Lack of Standardization and Legal Implications

Despite these efforts, substantial gaps in standardization persist. Variability in sampling sites, analytical methods, and interpretative criteria continues to limit reproducibility and comparability across studies and casework. This heterogeneity poses particular challenges in medico-legal settings, where the evidentiary value of microbiological findings may be scrutinized in court [12,50].

From a legal perspective, the absence of standardized protocols and validated interpretative frameworks may undermine the admissibility and weight of postmortem microbiological evidence. Addressing these gaps requires coordinated efforts among forensic pathologists, microbiologists, and legal experts.

## 8. Future Perspectives

In forensic practice, PMM rarely provides sufficient evidentiary weight when considered in isolation. PMM findings must therefore be interpreted within a multidisciplinary forensic framework that integrates microbiological data with pathological, toxicological, and contextual information. The value of PMM lies not in standalone diagnostic conclusions, but in its contribution to structured forensic reasoning.

Future progress in PMM will depend on balancing methodological innovation with forensic practicality. While advanced molecular techniques and AI-based tools offer promising research opportunities, their translation into routine practice requires rigorous validation, standardization, and clear interpretative criteria.

Priority areas for development include multicentric human studies, harmonized sampling and reporting protocols, and clearer definition of diagnostic thresholds and contextual requirements for reliable forensic interpretation.

## 9. Conclusions

PMM represents a valuable but inherently complex tool in forensic diagnostics. When applied selectively and interpreted with methodological rigor, PMM can significantly contribute to cause-of-death determination, particularly in infection-related and sudden unexpected deaths.

At the same time, postmortem microbial dynamics, contamination, and technological limitations impose substantial interpretative challenges. Advances in molecular biology and AI offer new opportunities, but their forensic use must remain cautious and evidence-based.

By acknowledging both its potential and its limitations, forensic practitioners can employ PMM as a meaningful component of modern forensic diagnostics, supporting informed forensic reasoning rather than replacing it.

## Figures and Tables

**Table 1:** Main applications of PMM in forensic diagnostics.

Forensic Scenario	Diagnostic Question	Main Samples	Analytical Methods	Diagnostic Value	Main Limitations
Suspected sepsis	Determination of an infectious cause of death	Blood, spleen, lung, liver	Culture, PCR, molecular assays	High when findings are concordant across multiple sites and supported by pathology	PMI, bacterial translocation, contamination
Central nervous system infections	Diagnosis of meningitis or encephalitis	Cerebrospinal fluid, brain tissue	Culture, PCR, molecular assays	High when correlated with histopathological findings	Sampling contamination, delayed sampling
Cardiac infections	Identification of infectious myocarditis	Heart tissue	PCR, molecular assays, histology	Moderate to high	Focal lesions, limited tissue involvement
Sudden unexpected death	Detection of occult or unrecognized infections	Multiple organs and fluids	Integrated microbiological and pathological approach	Variable	Interpretative complexity, lack of antemortem data
Vulnerable subjects (e.g., immunocompromised, splenectomized)	Identification of undiagnosed infections	Blood, spleen, target organs	Molecular methods, culture	High	Strong dependence on clinical and pathological context

**Table 2:** Major interpretation pitfalls in PMM.

Interpretation Pitfall	Underlying Mechanism	Diagnostic Risk	Mitigation Strategy
Postmortem microbial overgrowth	Loss of immune control and tissue autolysis after death promote uncontrolled microbial proliferation	False attribution of infection	Consider PMI and correlate with tissue inflammation and host response
Bacterial translocation	Breakdown of gastrointestinal and mucosal barriers leading to passive spread of enteric bacteria	False diagnosis of sepsis	Evaluate multi-site concordance and pathological evidence of systemic infection
Sampling contamination	Introduction of exogenous microorganisms during collection or handling	False-positive microbiological results	Strict aseptic techniques and standardized sampling protocols
Colonization by commensal flora	Presence of non-pathogenic or opportunistic microorganisms	Overinterpretation of causality	Assess anatomical plausibility and host inflammatory response
Molecular over-sensitivity	Detection of microbial DNA without evidence of viability or tissue invasion	Misleading interpretation of infection	Integrate molecular findings with culture results and supportive tissue changes

**Table 3:** Laboratory detection methods and computational analytical approaches used in postmortem microbiology (PMM).

	Main Strengths	Main Limitations	Forensic Applicability
Laboratory detection method			
**Culture-based methods**	Detection of viable microorganisms; possibility of antimicrobial susceptibility testing; low cost and wide laboratory availability	Reduced sensitivity after death; influenced by prior antimicrobial therapy; risk of overgrowth by commensal or contaminant flora	Suitable for routine forensic practice when supported by multi-site concordance and consistent postmortem findings
**Targeted PCR assays**	High analytical sensitivity; rapid detection of specific bacterial or viral pathogens, including fastidious organisms	Detection of microbial DNA does not imply viability or causality; contamination may lead to false-positive results	Useful for targeted diagnostic questions and as a complement to culture-based methods
**Broad-range 16S rRNA gene sequencing**	Broad, culture-independent detection of bacterial taxa; useful in culture-negative cases	Limited taxonomic resolution; inability to distinguish live from dead bacteria; complex interpretation	Adjunctive tool with mainly research-oriented applications in forensic diagnostics
**Shotgun metagenomic sequencing**	High taxonomic and functional resolution; simultaneous detection of bacteria, viruses, and fungi	High cost; complex bioinformatic analysis; susceptibility to environmental contamination	Experimental approach; currently not suitable for routine forensic casework
**Computational and analytical approach**			
**AI–based models**	Ability to identify complex patterns in large, high-dimensional datasets; potential support for classification tasks	Limited validation on human forensic cases; lack of transparency and interpretability; sensitivity to confounding variables	Exploratory tool; not appropriate as standalone forensic evidence

## Data Availability

No new data were created or analyzed in this study. Data sharing is not applicable to this article.

## Abbreviations

The following abbreviations are used in this manuscript:

PMBT	Postmortem bacterial translocation
PMM	Postmortem microbiology
PMI	Postmortem interval
AI	Artificial Intelligence
